# Comparative Evaluation of Phenoloxidase Activity in Different Larval Stages of Four Lepidopteran Pests After Exposure to *Bacillus thuringiensis*

**DOI:** 10.1673/031.012.8001

**Published:** 2012-07-12

**Authors:** J.A. Valadez-Lira, J.M. Alcocer-Gonzalez, G. Damas, G. Nuñez-Mejía, B. Oppert, C. Rodriguez-Padilla, P. Tamez-Guerra

**Affiliations:** ^1^ Departamento de Microbiología e Inmunologia, Facultad de Ciencias Biológicas, Universidad Autónoma de Nuevo León, México; ^2^ USDA-ARS, Center for Grain and Animal Health Research, 1515 College Ave., Manhattan, Kansas, USA

**Keywords:** *Heliothis virescens*, innate humoral response, *Plodia interpuctella*, *Spodoptera exigua*, *Trichoplusia ni*

## Abstract

Microbial entomopathogen—based bioinsecticides are recognized as alternatives to synthetic pesticides. Insects defend themselves against microbial pathogens by innate mechanisms, including increased phenoloxidase (PO) activity, but its relationship with microbial bioinsecticides efficacy is little known. This study evaluated the differences in PO activity at different developmental stages of the tobacco budworm *Heliothis virescens* Fabricius (Lepidoptera: Noctuidae), Indian meal moth *Plodia interpunctella* (Hübner) (Pyralidae), beet armyworm *Spodoptera exigua* (Hübner) (Noctuidae), and cabbage looper *Trichoplusia ni* (Hübner) (Noctuidae). Additionally, 2^nd^- and 4^th^-instars were exposed to the LC_50_ value of the commercial *Bacillus thuringiensis* (Bt) spray, Biobit^®^. The percentage of insecticidal activity (IA%) on 2^nd^-instar Biobit—exposed larvae was approximately the predicted 50 % mortality for all species except *S. exigua.* With all 4^th^ instar Biobit—exposed larvae, mortality was not significantly different from that of unexposed larvae. Unexposed insects had a significantly higher PO activity in pre—pupae and pupae than early—instar larvae and adults, whereas PO activity was higher in adult females than in males. Correlation analysis between IA% and PO activity revealed significant *r*—values (*p* < 0.01) in 2^nd^ instar *H. virescens* (*r* = 0.979) and *P. interpunctella* (*r* = 0.930). Second instar Biobit—exposed *P. interpunctella* had 10 times more PO activity than unexposed larvae. Similarly, the amount of total protein was lower in 4^th^ instar Biobit—exposed *H. virescens* and higher in *S. exigua.* Therefore, the results indicated a relationship between Biobit susceptibility and PO activity in some cases. This information may be useful if the Biobit application period is timed for a developmental stage with low PO activity. However, more studies are needed to determine the correlation of each insect with a particular bioinsecticide.

## Introduction

Food—borne microbes can be affected by enzymes of the digestive tract. Ingested organisms also may be harmed by the harsh pH and redox conditions in the alimentary canal. If microorganisms are successful in evading the passive immune responses, they still may encounter innate immune defenses (Stanley and Shapiro 2007). Arthropods rely on diverse mechanisms of immune response, both passive and innate. Innate immunity includes the prophenoloxidase (proPO) system, which is confined inside the hemocytes, and is manifested in a series of cascading enzymatic reactions by the stimulation of peptidoglycan, β—glucans or lipopolysaccharides, and phenoloxidase (PO) enzyme activation. proPO is activated to PO by serine proteases and is responsible for initiating the biosynthesis of quinones to melanin (Ashida and Dohke 1980; Bidla et al. 2009). Melanin is a brown—black pigment that inhibits entomopathogenic bacterial and fungal enzymatic activity by encapsulation, as has been observed in Lepidoptera (Jiang et al. 1998), and may be related to the efficacy of certain bioinsecticides.

Lepidopteran pests are controlled mainly through synthetic chemical insecticides, but the risk of ecological disturbance and resistance development has prompted research to identify better choices. One alternative to chemicals is the application of bioinsecticides, considered effective for the control of some lepidopteran pests. Among microbial entomopathogens, *Bacillus thuringiensis* (Bt) commercial products, and transgenic plants expressing one or more Bt toxins, are used worldwide. However, insects can defend against microbial pathogens by innate mechanisms including PO activity as part of the humoral response (Cerenius et al. 2008). More importantly, the immune response to biopesticides in arthropods may be related to their lack of efficacy, although it is unknown if innate responses are relevant to formulations containing only toxin proteins. In the present study, differences were evaluated in the innate immune response to Bt as PO activity in different life—cycle phases of four species including the tobacco budworm *Heliothis virescens* Fabricius (Lepidoptera: Noctuidae), Indian meal moth *Plodia interpunctella* (Hübner) (Pyralidae), beet armyworm *Spodoptera exigua* (Hübner) (Noctuidae), and cabbage looper *Trichoplusia ni* (Hübner) (Noctuidae). Two of these species, *P. interpunctata* and *T. ni* were selected because they have demonstrated differences in their susceptibility to Bt in our laboratory (Rubio-Cota et al. 2010; Tamez-Guerra et al. 2006). In addition, the insecticidal activity was compared among 2^nd^ and 4^th^ instar larvae that were either unexposed or exposed to the Bt commercial product Biobit^®^, to evaluate whether an insect immune response can affect susceptibility to Bt.

## Materials and Methods

### Insects

The, *S. exigua* and *H. virescens* colonies were established from field collected insects in Northeast Mexico in 2000, whereas the *T. ni* colony was obtained from Dr. Howard T. Dulmage (USDA-ARS, Weslaco, TX), and reared since 1982 in León on artificial diet as described in Tamez-Guerra et al. (2006). The *T. ni* colony has been crossed with field—collected Mexican populations every 5–7 years to avoid homocygamy—related problems. The *P. interpuctella* colonies were from the Center for Grain and Animal Health Research (Manhattan, KS) and reared in León on a previously described cracked wheat artificial diet (McGaughey and Beeman 1988). Insects were incubated at 25 ± 2 °C, 55–60 ± 10% RH, and 16:8 L:D photoperiod.

### Chemicals

All substrates and chemicals were from Sigma—Aldrich (www.sigmaaldrich.com) unless otherwise specified.

### Biobit insecticidal activity

Initial tests were conducted to determine the fifty percent lethal concentration (LC_50_) for insects exposed to Biobit HP 32,000 IU/mg potency (Valent Biosciences Corporation, www.valentbiosciences.com), produced from a Bt var. *kurstaki* strain from DuPont (www.dupont.com) using an overlayer bioassay (Tamez-Guerra et al. 2006). Bioassays were performed in triplicate by exposing 30 neonates of each insect to six Biobit concentrations, prepared as serial doses (diluted 1:2) in distilled water. For *H. virescens*, the highest concentration tested was 0.16 IU/cm^2^; for *T. ni*, 0.19 IU/cm^2^; and for *S. exigua*, 1.9 IU/cm^2^. 35 mL of each dose or distilled water only used as a control were applied to 5 mL wheat germ artificial diet, 7.1 cm^2^ surface area. Doses were air dried for 30 min and then infested with two neonates per cup with *S. exigua* or *T. ni*, or one per cup with *H. virescens.* The insecticidal activity of Biobit against *P. interpunctella* was determined using a diet-incorporated bioassay with six doses (0, 0.6, 1.2, 2.4, 4.8, 9.6, and 19.2 µg/g of diet) (McGaughey and Johnson 1992). For this assay Biobit doses were prepared by incorporating 1.5 mL of each Biobit dose into 5 g of wheat-germ diet and allowed to air dry, and then infesting with 10 *P. interpunctella* neonates in triplicate. Treatments were incubated in 14:10 L:D photoperiod at 28 °C. To calculate LC_50_ values for Biobit, mortality data for each lepidopteran species were evaluated after five days and analyzed using POLO-Plus (LeOra 2007).

### PO activity in unexposed and Biobit—exposed larvae

PO activity was measured from the hemolymph of different developmental stages of *P. interpunctella*, *H. virescens*, *S. exigua*, and *T. ni* using a technique first described by Ashida (1971) and Seed et al. (1978), and modified by Harizanova et al. (2004). The bioassay was conducted with 2^nd^ or the 4^th^ instar larvae of each lepidopteran species, using the LC_50_ values for Biobit-exposed neonates (Table 1), and using the overlayer bioassay for *H. virescens*, *S. exigua*, and *T. ni*, or the diet—incorporation bioassay for *P. interpunctella* as previously described. In these bioassays, 40 larvae of each insect species, either 2^nd^ or 4^th^ instar, were incubated for 24 hours on either the control or Biobit—treated diet; 20 larvae were used for insecticidal activity (IA) determination and the other 20 for phenoloxidase (PO) activity and protein analysis.

For PO activity and protein analyses, hemolymph was collected by gently removing an anterior proleg, using a 14 cm sterile entomological dissection scissor. Hemolymph was collected directly into a chilled 1.5 mL microcentrifuge tube on ice (Shelby and Popham 2006) and was diluted 1:24 with ice—cold PBS. Hemolymph was frozen for 48 hours to lyse the hemocytes and release the inner—cell plasma. Samples were thawed and centrifuged at 5000 rpm for 1 min to separate the plasma containing PO. 50 mL aliquots of plasma sample were placed in a microplate well, and 150 µL of 10 mM DL-DOPA were added to each well as substrate. PO activity was measured and calculated as previously described. Two hundred microliters of substrate 10 mM D*L*-dihydroxyphenylalanine (DL-DOPA) were added to each well. Microplates were incubated in the dark at room temperature, and absorbance was read at 490 nm every 5 min for 30 min, using a microplate reader (Multimode detector DTX 880, Beckman Coulter Inc., Austria). As a negative control, phosphate buffered saline (PBS, 1.48 g of Na_2_HPO_4_, 0.43 g of NaH_2_PO_4_, 7.2 g NaCl, 1000 mL distilled water, pH 7.2) with substrate only was monitored over the same time periods and was subtracted as background. PO-specific activity was defined as the change in optical density over time. Tests were in triplicate with insects from different rearing lots. Data were analyzed using ANOVA posthoc Tukey α = 0.05 SPSS version 17.0 (SPSS 2008). This bioassay was performed in triplicate.

**Table 1.**
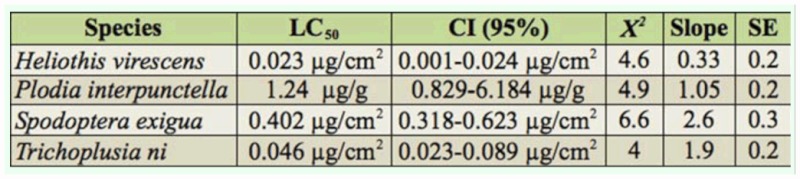
50% lethal concentrations (LC_50_) for Biobit in four lepidopteran pests.

Protein determination was performed in triplicate using the diluted hemolymph from the PO activity assay (Bradford 1976). A standard curve was prepared with standard concentrations of 12 serial BSA dilutions, from 0.0 to 2.0 mg/mL, using PBS as diluent. For treatment analysis, 5.0 µL of diluted hemolymph from each sample and 200 µL of Bradford reagent were mixed and transferred to a 96—well plate. Absorbance was read in a spectrophotometer (Beckman Coulter Inc.) at 595 nm 2–10 min after mixing. The reaction setting time was selected to allow adequate reaction development, but no longer than 10 min to prevent oxidation. Total protein was calculated by comparison to the standard curve value.

### Correlation analysis

Correlation analyses comparing insecticidal activity versus PO activity, total protein versus PO activity, or insecticidal activity versus total protein of each 2^nd^ and 4^th^ instar larvae from unexposed or Biobit—exposed insects was performed by using Pearson's analysis (SPSS 2008) using a cutoff for significance *p* < 0.05.

## Results

### Susceptibility to Biobit

Susceptibility to Biobit—exposed 2^nd^ or 4^th^ instar larvae showed that the most susceptible were *H. virescens* neonates, whereas the bioinsecticide was least toxic when tested against *P. interpunctella* neonates (Table 1). ANOVA comparison among all 24—hour Biobit—exposed 2^nd^ or 4^th^ instar larvae demonstrated a significantly higher IA in 2nd than 4th instar larvae (*p* ≤ 0.05, Table 2). The IA of Biobit in 2^nd^ instar *H. virescens*, *P. interpunctella*, and *T. ni* was close to the expected 50% mortality (50, 44, and 46%, respectively). However, 2^nd^ instar *S.* *exigua* larvae exposed to Biobit resulted in an IA of only 20.0%. IA among of all 4^th^ instar lepidopterans demonstrated a lack of susceptibility to Biobit, as survival was similar to that of unexposed larvae.

### Phenoloxidase activity in Biobit—treated Lepidoptera

PO activity in the hemolymph of Biobit—exposed larvae was compared to that of control (unexposed) larvae (Table 2). In general, PO activity was higher in *H. virescens* and lowest in *T. ni* larvae. Increased PO activity was found in 2^nd^ instar Biobit—exposed larvae, but the increase was significant only in *P. interpunctella* larvae. PO activity was highest in 4^th^ instar control *H. virescens* larvae, and significantly lower PO activity was found when larvae were exposed to Biobit.

**Table 2.**
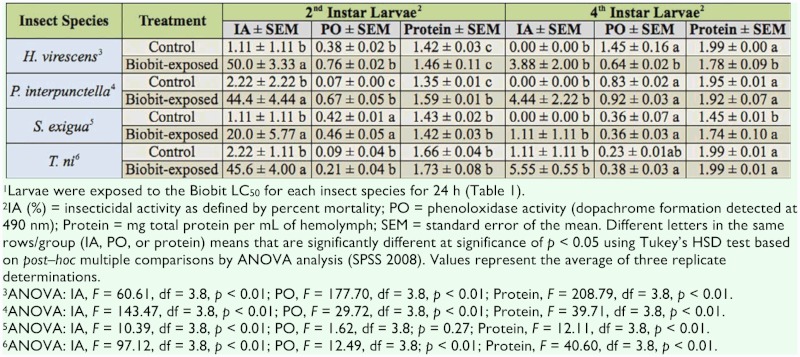
Insecticidal activity, phenoloxidase activity, and total protein (mg/mL) of 2^nd^ or 4^th^ instar *Heliothis virescens*, *Plodia interpunctella*, *Spodoptera exigua*, and *Trichoplusia ni* larvae, either control or exposed to Biobit for 24 hours. ^1^

### Total protein determination

Differences in total protein among different insect species or treatments were significant in some cases (Table 2). In general, protein values were higher in 4^th^ than in 2^nd^ instar larvae. In comparing treatment effects, the total protein was significantly more in 4^th^ instar *H. virescens* control than in Biobit—treated larvae. However, the total protein in Biobit—exposed 2^nd^ instar *P. interpunctella* and 4^th^ instar *S. exigua* larvae was significantly more than found in that of the respective control larvae.

### Correlation analysis

Three correlation analyses were performed: IA versus PO activity, IA versus total protein, and PO activity versus total protein (Table 3). IA versus PO activity correlation analysis resulted in a negative value only with 4^th^ instar *H. virescens* larvae (*r* = -0.677), but the correlation was not significant (*p* = 0.140). Significant positive correlation (*p* < 0.01) was observed in IA versus PO activity among 2^nd^ instar Biobit—exposed *H. virescens* (*p* < 0.01) and *P. interpunctella* (*p* < 0.01) larvae, as well as in 4^th^ instar Biobit—exposed *T. ni* larvae (*p* < 0.01). However, there was no significant correlation between IA and PO in Biobit—exposed *S. exigua* larvae.

**Table 3.**
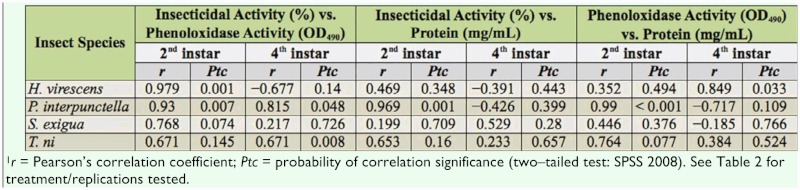
Correlation analysis of insecticidal activity, phenoloxidase activity, and protein (me/mL)^1^.

The correlation analysis of IA versus total protein was significant only in 2^nd^ instar Biobit—treated *P. interpunctella* (*p* < 0.01). There was no significant correlation of IA and total protein in *H. virescens*, *S. exigua,* and *T. ni* treated larvae.

Correlation analysis of PO activity versus total protein was significant in 4^th^ instar Biobit—exposed *H. virescens* larvae (*p* < 0.05). Similarly, PO and total protein were correlated in 2^nd^ instar Biobit—exposed *P. interpunctella* larvae (*p* < 0.01). No significant correlation of PO activity and total protein was observed in *T. ni* or *S. exigua.*

## Discussion

In the present study, we evaluated the relationship among Bt susceptibility and PO activity levels (as representative of the innate immune response) among laboratory colonies of four selected Lepidoptera. Samples included hemolymph from unexposed or Biobit-exposed 2^nd^- and 4^th^-instar larvae.

Of the species tested, *Heliothis virescens* was the most susceptible lepidopteran to Biobit, whereas the least susceptible was *P. interpunctella.* The percentage mortality of 2^nd^ instar larvae exposed to the LC_50_ value of Biobit was similar to the predicted 50% in all of the insects tested, except *S. exigua* in which mortality was only 20%. Although the product recommendation dose is similar for *H. virescens* and *S. exigua* (http://www.proagro.com.mx/prods/valent/valent02.htm), we observed that *H. virescens* susceptibility was 17-fold higher than that of *S. exigua* in the laboratory. According to previous bioassays, *H. virescens* and *T. ni* were more susceptible to Cry 1A and 2A toxins, *P. interpunctella* was susceptible only to 1A, and *S. exigua* was not susceptible to any of the these toxins (Table 4), whereas *P. interpunctella* was susceptible to Cry1A toxins (Cry toxins in Biobit are 1Aa, 1Ab, 1Ac, 2Aa, and 2Ab). The different bioassay method, in which the toxin was incorporated into the diet instead of concentrated on the surface, may have contributed at least in part to an increased LC_50_.

**Table 4.**
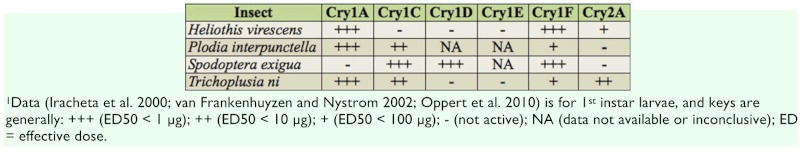
Relative sensitivity of *Heliothis virescens*, *Plodia interbunctella*, *Sbodobtera exigua*, and *Trichoblusia ni* to some *Bacillus* *thuringiensis* Cry toxins^1^.

In our study, Biobit was not effective on 4^th^ instar larvae. Similarly, Kwon and Kim (2008) reported that 5^th^ instar *S. exigua* larvae exposed either to Bt svar. *kurstaki* (Btk, Thuricide®) or to Bt svar. *aizawai* (Bta GB413, GreenBioTech Chungju, Korea) showed no differences in mortality compared with that of unexposed controls. However, if they applied the immune suppressor benzylideneacetone, mortality increased to 60 and 80% with Bta— and Btk, respectively. Previous reports have indicated that earlier stages in Lepidoptera were more susceptible to Bt toxins and viruses (Morris 1969; Huang et al. 1999; Fuxa et al. 1999). In our study, this was also true in all species except *S. exigua* (Figure 1). Increased specific activity of gut proteases was proposed to be associated with the larval age and the loss of Bt toxin sensitivity in 5^th^ instar *S. littoralis* larvae (Keller et al. 1996). The relationship of susceptibility to Bt and larval age has been studied in *P. interpunctella* (Nwanze et al. 1975) and *T. ni* (Engelhard and Volkman 1995). Nwanze et al. (1975) found that by using a dose of 25 mg/kg of whole wheat diet using Dipel^®^ in a diet-incorporation bioassay resulted in 100% mortality of 1st instar *P. interpunctella* larvae, whereas 200 mg/kg was needed to obtain the same mortality level in larvae 18–21 days old. *T. ni* exposed to *Autographa californica* multiple nucleocapsid nucleopolyhedrovirus (*Ac*MNPV) were more resistant as 4th instar larvae than earlier instars, probably due to their ability to completely clear the *Ac*MNPV infection from the midgut epithelium (Engelhard and Volkman, 1995). Similar results were observed with 4th instar *Lymantria dispar* larvae and the *Lymantria dispar* multiple nucleocapsid nucleopolyhedrovirus (*Ld*MNPV) compared to control (McNeil et al., 2010).

Popham et al. (2004) demonstrated that PO in the plasma of *H. virescens* might provide a constitutive, humoral innate antiviral immune response to *Helicoverpa zea* single capsid nucleopolyhedrovirus (*Hz*SNPY) infection. In our study, it was observed that the most Biobit—susceptible *H. virescens* larvae had lower levels of PO present in the hemolymph as one of the key enzymes of insect immune response, but it is also found in hemocytes of the cuticular matrix where it is involved in the molting process (Ashida and Brey 1995; Hillyer and Christensen 2005). In this regard, PO is involved in sclerotization of the cuticle and melanization associated with nodulation, encapsulation, and wound healing, and may provide cytotoxic quinonoid compounds to kill opportunistically invading microorganisms (Nappi and Christensen 2005). PO activation has been positively correlated to wounding or infection as part of the immune response (Kanost and Gorman 2008). PO activity was significantly lower in 2^nd^ instar *P. interpunctella* larvae compared with that of *H. virescens*, *S. exigua*, and *T. ni.* However, exposure to Biobit resulted in significantly increased PO activity only in 2^nd^ instar *P. interpunctella*, and may correlate to the relatively higher LC_50_ for this lepidopteran. Furthermore, the most Biobit—sensitive insect in our test, *H. virescens*, had significantly lower PO activity when 4^th^ instar larvae were exposed to Biobit. It was previously reported that PO levels increased in succeeding larval instars of *S. littoralis* and *P. interpunctella* (Ishaaya et al. 1974; Hartzer et al. 2005; Valadez-Lira et al. 2010). With *P. interpunctella* and *S. exigua*, an increase in immune response was related to decreased susceptibility to entomopathogens (Gassmann et al. 2009) and chemical insecticides (Liu et al. 2009). However, increased tolerance to Bt svar. *kurstaki* resulted in a reduced immune response and lower PO activity in susceptible—resistant *T. ni* (Ericsson et al. 2009) and increased in Biobit—exposed 2nd instar *P. interpunctella* and 4th instar *S. exigua* larvae, whereas total protein was lower in Biobit—exposed 4th instar *H. virescens* larvae.

In general, the total protein calculated value was higher among 4^th^ instar compared with 2^nd^ instar larvae, and was increased in Biobit—exposed 2^nd^ instar *P. interpunctella* and 4^th^ instar *S. exigua* larvae, whereas total protein was lower in Biobit—exposed 4^th^ instar *H. virescens* larvae.

Certainly, the major factors associated with sensitivity to Bt toxins have been previously characterized as toxin receptors and protoxin activation/solubilization (reviewed in Ferré and Van-Rie 2002). We found that IA and PO activity were positively correlated in Biobit—treated 2^nd^ instar *H. virescens* and *P. interpunctella* and 4^th^ instar *T. ni* larvae, suggesting that PO activity may contribute to the efficacy of Bt toxins. If PO is a factor in toxicity, elucidation of the PO levels in different developmental stages of lepidopteran pests may be used to enhance bioinsecticide performance in pest management strategies, particularly if application time is programmed accordingly.

Overall, our results may help to understand why bioinsecticides are more effective when applied to earlier instars in some insects, and may be useful as a tool to improve bioinsecticide efficacy and lead to further understanding of the mechanisms of innate immunity. Using PO activity as a physiological parameter may also help to determine immune response activation against entomopathogenic microbial infections (Narayanan 2004). Our results suggest that PO protects insects from microbial infection more effectively during later instars, but more studies are needed to determine the relationship between PO activity and susceptibility of an insect to a particular entomopathogen.

## **Abbreviations**

IA: insecticidal activity
PO: phenoloxidase
